# Effect of Distigmasterol-Modified Acylglycerols on the Fluidity and Phase Transition of Lipid Model Membranes

**DOI:** 10.3390/membranes12111054

**Published:** 2022-10-27

**Authors:** Hanna Pruchnik, Aleksandra Włoch, Witold Gładkowski, Aleksandra Grudniewska, Anna Chojnacka, Mateusz Krzemiński, Magdalena Rudzińska

**Affiliations:** 1 Department of Physics and Biophysics, Wrocław University of Environmental and Life Sciences, Norwida 25, 50-375 Wrocław, Poland; 2 Department of Food Chemistry and Biocatalysis, Wrocław University of Environmental and Life Sciences, Norwida 25, 50-375 Wrocław, Poland; 3 Faculty of Food Science and Nutrition, Poznań University of Life Sciences, 60-637 Poznań, Poland

**Keywords:** stigmasterol, acylglycerols, DPPC, DMPC, model lipid membrane, liposomes, fluidity, main phase transition, calorimetry, spectroscopy

## Abstract

Plant sterols are known for their health-promoting effects, lowering blood cholesterol levels and alleviating cardiovascular disease. In this work, we continue our research on the asymmetric acylglycerols in which fatty acid residues are replaced by two stigmasterol residues in *sn*-1 and *sn*-2 or *sn*-2 and *sn*-3 positions as new thermostable carriers of phytosterols for their potential application in foods or as components of new liposomes in the pharmaceutical industry. The aim of this manuscript was to compare and analyze the effects of four distigmasterol-modified acylglycerols (dStigMAs) on the fluidity and the main phase transition temperature of the model phospholipid membrane. Their properties were determined using differential scanning calorimetry (DSC), steady-state fluorimetry and attenuated total reflectance Fourier-transform infrared spectroscopy (ATR-FTIR). The determination of the effect of the tested compounds on the mentioned physicochemical parameters of the model membranes will allow for the determination of their properties and stability, which is essential for their practical application. The results indicated that all compounds effect on the physicochemical properties of the model membrane. The degree of these changes depends on the structure of the compound, especially the type of linker by which stigmasterol is attached to the glycerol backbone, as well as on the type of hydrocarbon chain.

## 1. Introduction

Phytosterols are bioactive compounds that make up the cell membrane of plants; one of their most well-known properties is their ability to lower cholesterol levels. They are found mainly in soybeans, vegetable oils and rice. They are also found in smaller quantities in fruits, vegetables and some species of nuts. The most familiar and widespread phytosterol in the human diet is stigmasterol (stigmasta-5,22-dien-3β-ol) [1], known for its cholesterol-lowering properties. As natural antagonists, phytosterols resemble cholesterol in structure, as well as function in cell membrane composition. Phytosterols, due to their valuable properties such as anti-inflammatory, anticancer, antioxidant, antidiabetic, antiatherogenic, are extensively used as functional ingredients in foods, pharmaceuticals, cosmetics and nutraceuticals [2,3,4,5,6,7,8]. Unfortunately, phytosterols found in foods are not resistant to damage during thermal processing or prolonged storage. The compounds formed during these processes exhibit proatherogenic and pro-inflammatory properties [9,10]. Hence, in order to reduce the formation of unhealthy compounds and to increase bioavailability, new sterol derivatives are being synthesized [11,12,13,14].

The subject of the research described in this paper are distigmasterol-modified acylglycerols (dStigMAs). Two stigmasterol molecules were attached to 3-palmitoyl-*sn*-glycerol in *sn*-1 and *sn*-2 position and to 1-oleoyl-*sn*-glycerol in *sn*-2 and *sn*-3 position [14]. Acylglycerols are used as carriers of various bioactive compounds (e.g., carotenoids or non-steroidal inflammatory agents), so they can also be an effective stigmasterol delivery system [15]. We used two different linkers between stigmasterol and acylglycerol (succinyl and carbonate) which yielded four compounds with different chemical structures and biological properties. In an earlier work, the stability and cytotoxic properties of these compounds were determined [10,14]. It was shown that the thermooxidative stability of the distigmasterol-modified acylglycerols was higher in comparison with free stigmasterol and its esters with palmitic and oleic acids [10]. In particular, dStigMAs were characterized by high thermooxidative stability. It was found that the type of linker connecting stigmasterol to acylglycerol and the heating temperature affected the cytotoxicity and genotoxicity of the obtained dStigMAs [10]. Potent cytotoxicity was exhibited by compounds with carbonate linker unlike to the compounds with succinyl linker which had no cytotoxic activity against normal human cells at 100 μg/mL and lower concentrations [14].

In this paper we report the interaction of dStigMAs with phospholipid model membranes. The objects of our study were model membranes (liposomes) formed from a mixture of phospholipid (DPPC, dipalmitoylphosphatidylcholine, or DMPC, dimyristoylphosphatidylcholine) and the tested compound at the following specified compound/phospholipid molar ratios: 0.20; 0.10; 0.05; 0.02 and 0.01. DPPC and DMPC are among the most abundant representatives of the group of biological membrane lipids and therefore they are very often used as the models for a lipid biomembrane [15,16,17,18,19]. Multilamellar liposomes were prepared for calorimetric studies, while unilamellar vesicles were prepared for fluorimetric investigations. Based on the studies, we were able to determine the impacts of dStigMAs on the fluidity of the phosphatidylcholine model membrane and on the phase transformations, including the temperature of the main phase transition of lipid. To the best of our knowledge, there are no studies on the effects of distigmasterol-modified acylglycerols on the fluidity of lipid model membranes. The results obtained will be helpful in the design of potential dietary supplements, such as liposomes containing stigmasterol as a health-promoting agent.

## 2. Materials and Methods

### 2.1. Materials

**Figure 1:**
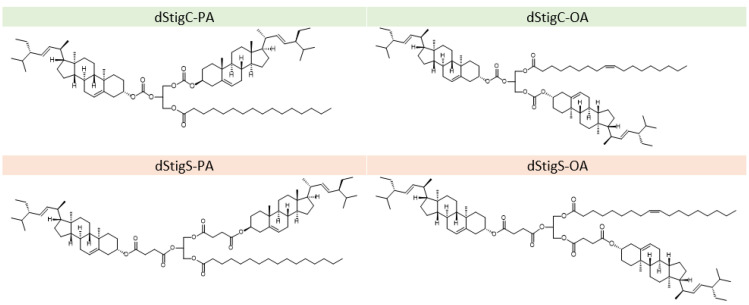
Structures of the studied compounds (dStigMAs): 1,2-distigmasterylcarbonoyl-3-palmitoyl-*sn*-glycerol (dStigC-PA); 2,3-distigmasterylcarbonoyl-1-oleoyl-*sn*-glycerol (dStigC-OA); 1,2-distigmasterylsuccinoyl-3-palmitoyl-*sn*-glycerol (dStigS-PA); 2,3-distigmasterylsuccinoyl-1-oleoyl-*sn*-glycerol (dStigS-OA).

Distigmasterol-modified acylglycerols (dStigMAs) were obtained in the Department of Food Chemistry and Biocatalysis, Wrocław University of Environmental and Life Sciences. Both 1,2-distigmasterylcarbonoyl-3-palmitoyl-*sn*-glycerol (dStigC-PA) and 1,2-distigmasterylsuccinoyl-3-palmitoyl-*sn*-glycerol (dStigS-PA) were synthesized from commercially available 3-palmitoyl-*sn*-glycerol. 2,3-Distigmasterylcarbonoyl-1-oleoyl-*sn*-glycerol (dStigC-OA) and 2,3-distigmasterylsuccinoyl-1-oleoyl-*sn*-glycerol (dStigS-OA) were synthesized from commercial 1-oleoyl-*sn*-glycerol. The synthesis and the structure of these compounds (Figure 1) have been described previously [14].

The fluorescent probe 1,6-diphenyl-1,3,5-hexatriene (DPH) was purchased from Molecular Probes (Eugene, OR, USA). The lipids 1,2-dimirystoyl-*sn*-glycero-3-phosphatidylcholine (DMPC) and 1,2-dipalmitoyl-*sn*-glycero-3-phosphatidylcholine (DPPC) were purchased from Sigma-Aldrich (St. Louis, MO, USA).

### 2.2. Lipid Model Membrane Preparation

Multilamellar (MLVs) and small unilamellar (SUVs) vesicles were used to study the effect of modified acylglycerols on the fluidity and thermotropic parameters of a model lipid membrane. We chose DPPC and DMPC for formulation because phospholipids are the most common class of lipids in biological membranes. Moreover, these types of models are well described in the literature. Liposomes were formed by the standard thin lipid film hydration method [15,17,19]. All lipids and tested compounds were dissolved with chloroform. Solutions of dStigMAs compounds were added to the lipid solution (DMPC or DPPC) at the appropriate concentration. The molar ratio of modified acylglycerols to phospholipids(dStigMAs/DPPC or dStigMAs/DMPC) was 0.01, 0.02, 0.05, 0.10 and 0.20, respectively. Samples were evaporated with a stream of nitrogen to a dry film, and then left in a vacuum pump for two hours. Such a dry lipid film, formed on the walls of the vessel after evaporation of the solvent, has a layered structure, identical to a bilayer. During the hydration of the film, water molecules explore it, penetrate between the individual bilayers, causing them to expand, separate and then “bud”. The resulting lipid film was eluted from the tube wall with redistilled water. The whole was shaken mechanically for about 15 min until a homogeneous milky suspension was obtained. Vesicles due to gentle shaking break off, and as they were formed from a multilayer lipid film, they are MLVs. The elution and shaking took place at a temperature above the temperature of the main phase transition of the lipid. Then, to obtain small liposomes (SUVs) ranging in size from 120 to 140 nm, the multilamellar vesicles were disrupted with ultrasound using a Sonics VCX750 sonicator (Sonics, Newtown, CT, USA for 900 s at 20 kHz. For the SUVs, the obtained sizes were 100–120 nm. The measurement was performed using Zetasizer Nano ZS (Malvern Panalytical, UK).

### 2.3. Differential Scanning Calorimetry (DSC)

MLVs were used as model membranes for DSC measurements. A detailed description of the method is provided in our previous work [20,21]. Preparation description of the liposomes is provided in Section 2.2. A suspension of multilayer liposomes (DMPCs or DPPCs) and liposomes with a compound (dStigMAs/DMPC or dStigMAs/DPPC) was placed in 40 μL aluminum calorimetric crucibles. The crucibles, using a suitable press, were closed tightly. The molar ratios of compound to DMPC or DPPC lipids were 0.01, 0.02, 0.05, 0.10 and 0.20, respectively. Samples were stored for 48 h at 4 °C until measured. Measurements were carried out using a Mettler Toledo Thermal Analysis System D.S.C. 821e° calorimeter. Depending on the type of lipid, the measurement range was 10 to 30 °C for DMPC, or 25 to 50 °C for DPPC. The heating rate was the same, 2 °C min^−1^. Measurements were repeated three times. The temperature of the pre-transition (T_p_) and main phase transition (T_m_), the peak half-width (ΔT_1/2_) and the enthalpy change (ΔH) of the main phase transition were analyzed using the original Mettler Toledo software (STAR^e^ SW 12.10, Mettler-Toledo, LLC, Columbus, OH, USA).

### 2.4. Steady-State Fluorescence Spectroscopy

SUVs were used as model membranes for fluorometric measurements. A detailed description of the method is provided in our previous work [19,22]. A detailed description of the preparation of the liposomes is provided in Section 2.2. A hydrophobic DPH dye was used to study the fluidity and temperature changes of the main lipid membrane phase transition. The fluorescence probe was added to unilamellar liposome, the final concentration of DPH in each sample was 1 µM. The samples, control (SUVs formed from DPPC or DMPC) and with the tested compounds (dStigMAs/DPPC or dStigMAs/DMPC) were then incubated without light at room temperature for a minimum of 30 min. Measurements were made in quartz cuvettes using a CARY Eclipse fluorimeter (Varian, San Diego, CA, USA) with a DBS Peltier module for sample temperature control. Measurements for all probes were taken at three temperatures: 25 °C (lamellar gel phase of DPPC), 37 °C (ripple phase of DPPC) and at 45 °C (lamellar, liquid crystal phase of DPPC). Additionally, for n_dStigMAs_/n_DPPC_ = 0.05 and n_dStigMA_s/n_DPPC_ = 0.20, a temperature scan was performed in two-degree increments between 25 and 50 ℃; for n_dStigMAs_/n_DMPC_ = 0.05 and n_dStigMA_s/n_DMPC_ = 0.20, a temperature scan was performed in two-degree increments between 14 and 35 ℃. The excitation wavelength for the DPH probe is λ_exc_ = 360 nm, while the emission wavelength is λ_em_ = 426 nm. Measurements were repeated three times.

Based on the changes in DPH intensity under polarized light, the anisotropy value (A) was determined according to the formula used in our previous publications [21,23].

### 2.5. Attenuated Total Reflectance Fourier Transform Infrared Spectroscopy (ATR-FTIR)

Fourier’s attenuated total reflection infrared spectroscopy was used to study the structure and fluidity of the phospholipid bilayer [17]. Measurements were made using a Nicolet 6700 FT-IR spectrometer (Thermo Fisher Scientific, Waltham, MA, USA). Analysis of the resulting spectra was performed using OMNIC (Thermo Nicolet) software. All measurements were performed in triplicate on two plates (ZnSe crystal). A background measurement (pure crystal) was performed for both plates, as well as a control test for distilled water alone. To the lipids (DPPC or DMPC) dissolved in chloroform, the dStigMAs were added at a concentration such that the molar ratio of the compound to the lipid was 0.02. They were then applied to the crystals of the two plates and evaporated with a stream of nitrogen. To obtain a dry lipid film free of organic solvent, the plates were left under vacuum for two hours. Then they proceeded to hydrate the phospholipid film, which was achieved by adding distilled water at a temperature above the main DMPC (or DPPC) phase transition. The test samples prepared in this way were sealed and left for 48 h, after incubation measurements were taken at eight temperatures (14, 16, 18, 20, 22, 24, 26, 28 °C and 35 °C for DMPC or 25, 30, 34, 36, 38, 40, 42, 45°C and 50 °C for DPPC) using a thermostat (Thermo Scientific). Each spectrum measurement at a constant temperature was performed three times. Each single spectrum was obtained from 128 records at 2 cm^−1^ resolution in the range 700–4000 cm^−1^.

## 3. Results

### 3.1. Differential Scanning Calorimetry (DSC)

**Figure 2:**
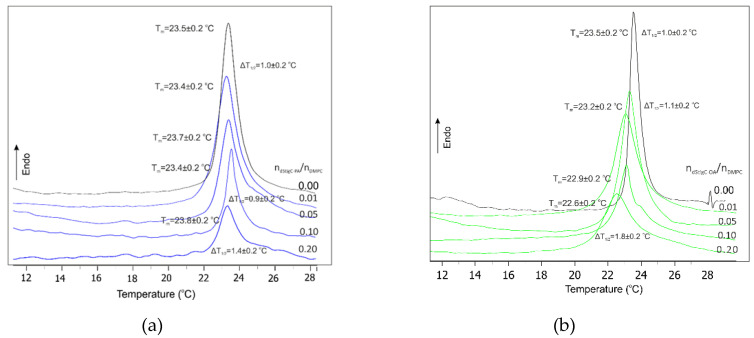
(**a**) Calorimetric curves of DMPC and dStigC-PA/DMPC multilamellar liposomes; (**b**) calorimetric curves of DMPC and dStigC-OA/DMPC multilamellar liposomes.

**Figure 3:**
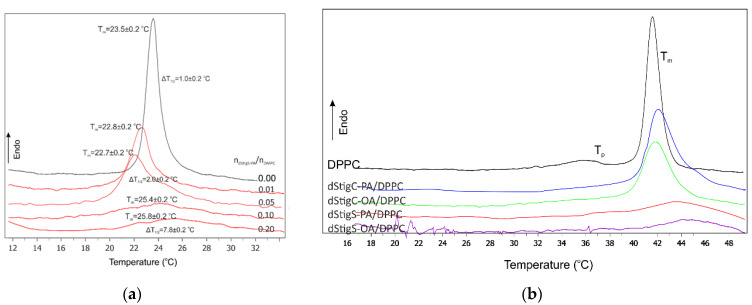
(**a**) Calorimetric curves of DMPC and dStigS-OA/DMPC multilamellar liposomes; (**b**) calorimetric curves of DPPC and dStigMAs/DPPC multilamellar liposomes (molar ratio of dStigMAs /DPPC = 0.2).

We used DSC to study the influence of distigmasterol-modified acylglycerols on the phase transition and structure of DMPC and DPPC model membranes, because this technique allows for the direct measurement of the thermodynamic parameters of the system. From the thermogram, several parameters characterizing this transition can be determined. First of all, the temperature of the main phase transition, denoted in Figure 2, Figure 3 and Figure S1 as T_m_, is the temperature for which the differential specific heat absorbed by the system reaches a maximum. For a symmetric curve, T_m_ represents the temperature for which the transition from the low-temperature state (gel) to the high-temperature state (liquid crystal) is halfway accomplished (Figure 2a,b and Figure 3a). However, phospholipid single- and multicomponent model membranes often have asymmetric thermograms, and then T_m_ and the temperature of the center of the phase transition do not coincide. From the thermogram, one can infer the cooperativity of the phase transition, which is closely related to the sharpness of the heat absorption curve. This sharpness is often expressed by the value of the temperature interval in the middle of the height of the absorption maximum and denotes ΔT_1/2_ (half-width). For some lipids, including DPPC, in addition to the two phases of gel and liquid crystal, there is an intermediate phase, the so-called ripple phase. We can determine one more parameter, the pretransition temperature (T_p_) from the lamellar gel phase to the ripple phase—Figure 3b. In the case of model membranes formed from pure lipids, two characteristic peak transitions were observed; according to literature data, pretransition and main phase transition (Figure 3b). The temperature of the main phase transition is characteristic for individual lipids, and for DPPC is about 42 °C, and for DMPC about 24 °C. The introduction of an additional compound into the membrane can change the thermotropic parameters of the system, such as melting temperature, enthalpy changes and half-width of the main peak transition. Introduced compounds can reduce the mobility of alkyl chains and thus change the temperature of the main phase transition. The effect of selected dStigMAs on the main phase transition of DMPC is shown in Figure 2 and Figure 3a. All distigmasterol-modified acylglycerols cause the elimination of the pretransition, reduce the cooperativity of the main phase transition and affect the temperature of the main phase transition. The degree of these changes depends on the structure of the compound; interestingly, rather than a chain, the type of linker has a greater effect on the fluidity of the lipid bilayer (Figure 2b and Figure 3a). However, the type of acyl chain also affects the changes in the temperature of mixed bilayers; the presence of one double bond causes a significant reduction in the temperature of the main phase transition, which is particularly evident for the compound dStigC-OA. In the case of dStigS-OA, we observe a slight decrease in the T_m_ at the lower concentration of compound in the membrane (n_dStigS-OA_/n_DPPC_ = 0.01 and 0.05), increase of the concentration causes the disappearance of the pronounced phase transition and significantly increases the width of the half peak. Figure 3b and Figure S1 summarize the calorimetric curves measured for the highest concentration of the tested compounds in the membrane: n_dStigMAs_/n_DPPC_ = 0.20 and for n_dStigMAs_/n_DMPC_ = 0.20. The analysis of the DSC curves shows that dStigS-PA and dStigS-OA induce slightly different changes of thermotropic parameters in comparison with dStigC-PA and dStigC-OA. The analysis of the DSC curves shows that dStigS-PA and dStigS-OA induce slightly different changes of thermotropic parameters in comparison with dStigC-PA and dStigC-OA. As the concentration of the compounds increases, the T_m_ shifts toward higher values, the enthalpy and ΔT_1/2_ of the transition decrease significantly, and the cooperativity of the transition decreases significantly, which may indicate a change in the fluidity of the lipid membrane. An increase in the concentration of dStigMAs, in particular of acylglycerols containing stigmasterol attached by succinyl linker, in the lipid bilayer leads to the disappearance of the main phase transition.

A shift in the temperature of the main phase transition toward lower values is observed mainly for dStigS-OA and dStigC-OA, i.e., compounds containing oleic acid, which is associated with a lower ordering in the gel phase of the acyl chains and a slight reduction in the temperature of the main phase transition. In addition, the asymmetric shape of the transition suggests the formation of domains containing an unbalanced amount of dStigMAs. A key role is played by stigmasterol, which acts as a separator by reducing the interactions of acyl chains of phospholipids. An increase in the concentration of compounds results in an increase in the content of stigmasterol molecules, the presence of which reduces van der Waals interactions between chains, resulting in a decrease in the enthalpy of the transition. An increase in the concentration of compounds and thus stigmasterol causes the disappearance of the sharp phase transition. In the liquid crystalline state, stigmasterol causes a decrease in the average surface area per lipid near the membrane surface.

### 3.2. Steady-State Fluorescence Spectroscopy

The influence of acylglycerols containing succinyl linker (dStigS) and carbonate linker (dStigC) on the membrane fluidity and temperature of the main phase transition of DPPC and DMPC was determined by the fluorimetric method using the DPH probe on the basis of changes in the anisotropy (A) of DPH fluorescence. The dye chosen for the study has an affinity for hydrocarbon chains and is located in the hydrophobic region of the lipid bilayer.

**Figure 4:**
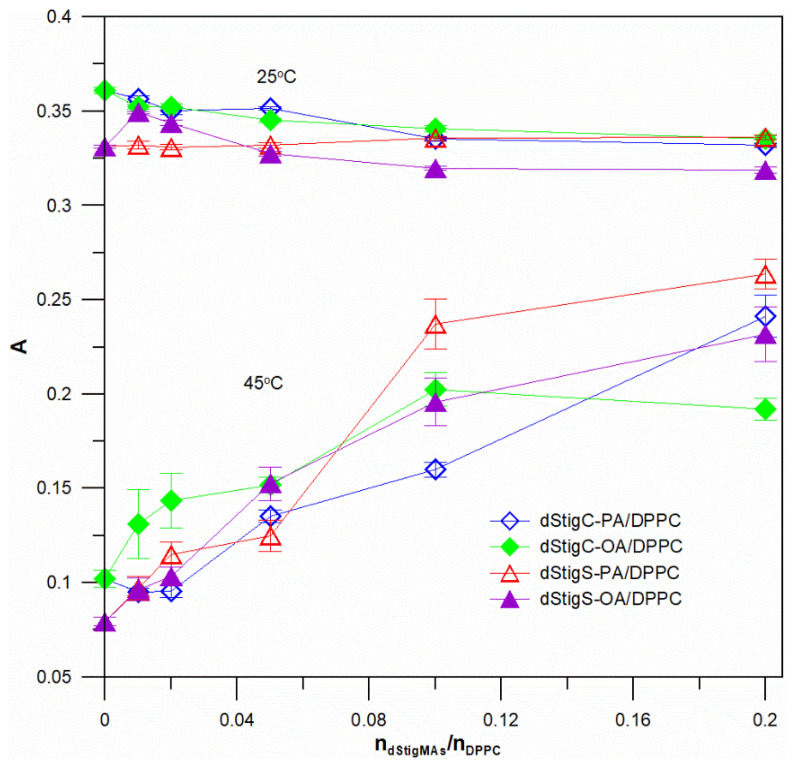
Values of fluorescence anisotropy of the DPH probe for different concentrations of the tested compound in the lipid membrane. Measurements made at two temperatures 25 and 45 °C for SUVs formed from DPPC and dStigMAs.

**Figure 5:**
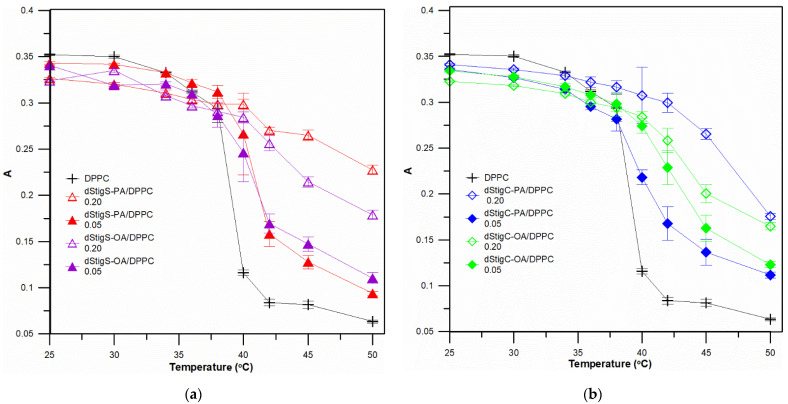
Values of anisotropy of DPH probe fluorescence as a function of temperature. (**a**) Measurements made for SUVs formed with DPPC, without and in the presence of dStigS-PA or dStigS-OA, for two molar ratios of dStigMAs /DPPC: 0.05 and 0.20. (**b**) Measurements made for SUVs formed with DPPC, without and in the presence of dStigC-PA or dStigC-OA, for two molar ratios of dStigMAs/DPPC: 0.05 and 0.20.

**Figure 6:**
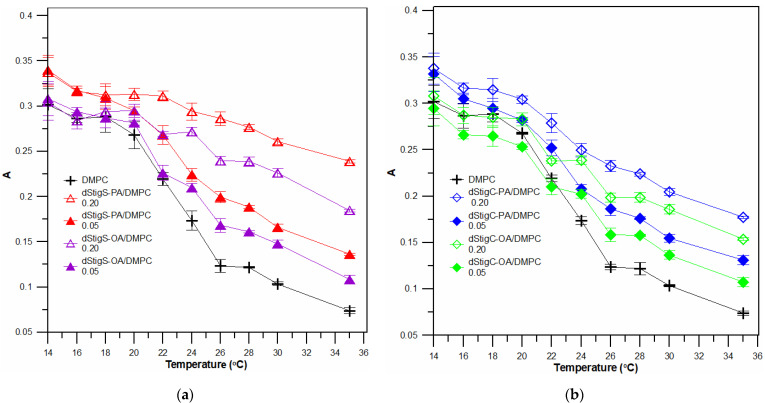
Values of anisotropy of DPH probe fluorescence as a function of temperature. (**a**) Measurements made for SUVs formed with DMPC, without and in the presence of dStigS-PA or dStigS-OA, for two molar ratios of dStigMAs /DMPC: 0.05 and 0.20. (**b**) Measurements made for SUVs formed with DMPC, without and in the presence of dStigC-PA or dStigC-OA, for two molar ratios of dStigMAs/DMPC: 0.05 and 0.20.

Measurements were performed at different temperatures, below and above the temperature of the main phase transition of the lipid, i.e., in the gel phase and in the liquid crystalline phase of the lipid membrane. Figure 4 shows example measurements at two temperatures of fluorescence anisotropy for DPH probe localized in bilayer of liposomes formed from DPPC and dStigMAs. At the temperature 25 °C, a slight increase in membrane fluidity (a slight decrease in anisotropy values for selected compounds) was observed, and in the liquid crystalline phase (45 °C) a significant increase in anisotropy values was observed depending on the value of the molar ratio of n_dStigMA_/n_DPPC_ for all distigmasterol-modified acylglycerols. For selected molar ratios of dStigMAs and DPPC (or DMPC), measurements were performed at several temperatures: 25, 30, 34, 36, 38, 40, 42, 45, 50 °C (or 14, 16, 18, 20, 22, 24, 26, 28 and 35 °C). Figure 5 shows the dependence of fluorescence anisotropy as a function of temperature for two molar ratios of n_dStigMAs_/n_DPPC_ (0.20 and 0.05). All compounds, although not to the same extent, cause changes in T_m_. The transition from the gel phase to the crystalline phase is characteristic of DPPC changes. In the presence of dStigMAs, rapid chain melting is not observed, the phase transition occurs over a wider temperature range, becomes blurred, and T_m_ shifts toward higher values. The results obtained are consistent with the DSC results. Below T_m_, the addition of all distigmasterol-modified acylglycerols has a decreasing effect on the anisotropy value, while above it, the A value increases. It was found that in the gel phase, the presence of both dStigC and dStigS causes a decrease in anisotropy values, and an increase in the liquid crystalline phase compared to pure DPPC. However, slightly larger changes in anisotropy in the liquid crystalline phase are observed for dStigS. As shown in Figure 5, the fluidity of the bilayer is affected not only by the linker, but also by the type of chain. The effect of chain type on membrane fluidity is also very apparent for liposomes formed from DMPC and dStigMAs. Figure 6a compares dStigS compounds containing a palmitic and oleic chain, and Figure 6b compares dStigC compounds. Similarly, as for membranes formed from DPPC at the highest concentration of dStigS-PA tested, we observe the absence of separation between the gel and liquid crystalline phases. The effect is slightly smaller for dStigS-PA. The presence of dStigC in the DMPC membrane shifts T_m_ toward higher values, but does not cancel the main phase transition. Interestingly, in the case of liposomes formed from DMPC, in contrast to DPPC, the studied compounds practically do not reduce the value of anisotropy below the temperature of the main lipid phase transition (Figure 6). 

Higher values of anisotropy indicate that the movement of the DPH molecule is more restricted, hence a more ordered structure of the bilayer can be assumed. Therefore, these results may indicate that in the membrane in the gel phase, the addition of sterols may introduce disorder, facilitating the movement of probe molecules in the membrane of DPPC. Conversely, in the membrane in the liquid crystalline phase, above the main DPPC (or DMPC) phase transition, dStigMAs can produce a more ordered membrane. An increase in the value of fluorescence anisotropy compared to the control indicates an increase in membrane rigidity (reduced tracer mobility), while a decrease in this value indicates an increase in membrane fluidity (an increase in tracer mobility in the membrane is associated with an increase in the mobility of lipid alkyl chains). Thus, we observe a similar but not identical effect as for cholesterol and stigmasterol [24,25,26] with higher concentrations of distigmasterol-modified acylglycerols, the sharp phase transition disappears, the membrane fluidity slightly increases in the gel phase and decreases in the liquid crystalline phase, the effect of these changes is more pronounced for dStigS. The degree of changes in membrane fluidity depends on the structure of compound, the compound that causes a significant increase in the “stiffness” of the membrane in the liquid crystalline phase, above the temperature of the main phase transition of DPPC (or DMPC) is dStigS-PA, followed by dStigS-OA.

### 3.3. Attenuated Total Reflectance Fourier Transform Infrared Spectroscopy (ATR-FTIR)

DSC is the primary method used to measure the thermodynamic parameters of lipid phase transitions, while spectroscopy techniques provide detailed information about lipid structure and dynamics at the molecular level. The IR was used to check the effect of dStigMAs on phase transitions of DMPC and of DPPC. For infrared spectra of phospholipids, it is possible to distinguish characteristic areas, the hydrophobic one coming from molecular vibrations of hydrocarbon chains, the interface and polar groups of lipids. In our work, we mainly focused on the hydrophobic region. We analyzed the effect of distigmasterol-modified acylglycerols on the vibrations of DMPC (or DPPC) hydrocarbon chains at different system temperatures, below and above the DMPC (or DPPC) main phase transition temperature.

Stretching vibrations of hydrophobic hydrocarbon chains, i.e., methylene and methyl groups, occur in the region from 3100 to 2800 cm^−1^. The stretching vibration bands of the methylene group ν(CH_2_), are characterized by the highest intensity among all phospholipid bands and lie in the range 2916–2923 cm^−1^ and 2849–2854 cm^−1^ for the asymmetric ν_as_(CH_2_) and symmetric ν_s_(CH_2_) vibration, respectively. The position of the maxima of these bands changes depending on the ordering of the lipids. Conformational changes in the hydrocarbon chains of lipids are influenced either by the temperature of the system or by substances present in the bilayer. The frequency values are lower when the hydrocarbon chains are in an all-*trans* conformation and higher when disorder increases, indicating an increase in *gauche* rotamers in the acyl chains [27].

**Figure 7:**
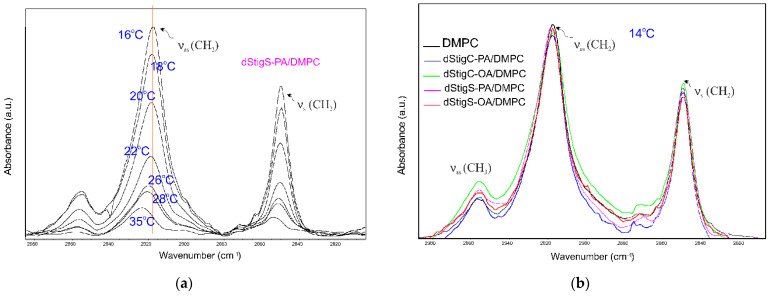
Infrared spectra of hydrated dStigS-PA/DMPC bilayer in the hydrocarbon chains region at different temperatures (**a**) and comparison of the spectra from the hydrophobic region of the bilayer of all tested compounds at the same temperature, in the gel phase (**b**); n_dStigMAs_/n_DMPC_ = 0.20.

**Figure 8:**
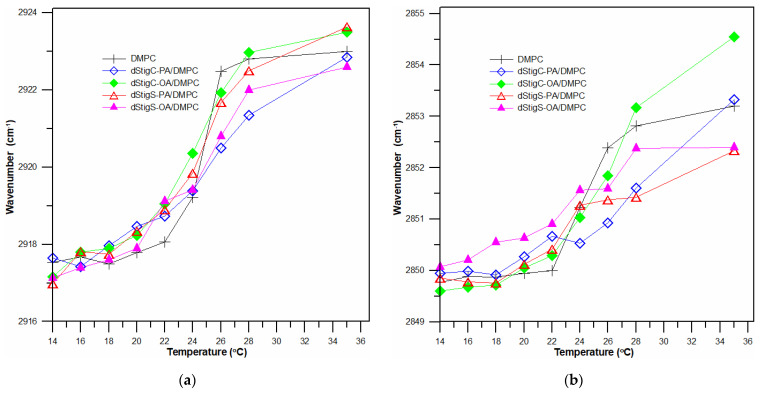
Dependence of vibration frequency on system temperature of the CH_2_ asymmetric (**a**) and symmetric (**b**) stretching mode in the presence and absence of dStigMAs for DMPC bilayer; n_dStigMAs_/n_DMPC_ = 0.20.

Selected ATR-IR spectra of hydrated dStigS-PA/DMPC bilayer in the hydrocarbon chains region at different temperatures are shown in Figure 7a. Figure 7b compares the spectra from the hydrophobic region of the bilayer of all tested compounds at the same temperature, in the gel phase. The frequency of both symmetric and asymmetric oscillations in the presence of the tested compounds in the DMPC membrane is slightly shifted. These shifts vary depending on the structure of the compound, the presence of compounds with oleic acid (dStigC-OA) above the temperature of main phase transition causes slight shifts toward higher values of wave numbers, while for a compound with palmitic acid we observe slight shifts toward lower values (dStigC-PA). Figure 8 compares the temperature-dependent changes in the vibration frequency of hydrocarbon chains for DMPC and for the dStigMAs/DMPC system. DMPC shows a sharp increase in frequency at around 24 °C, which indicates a cooperative phase transition, where there is a melting of the hydrocarbon chains. Interestingly, for dStigC/DMPC and dStigS/DMPC systems, greater changes compared to DMPC are observed for CH_2_ symmetric stretching vibrations. The phase transition becomes wider, the sharp separation between the gel and liquid crystal phases disappears, especially for dStigS compounds. The phase transition becomes wider, which was also observed in DSC measurements. The frequency of the asymmetric CH_2_ stretching band at temperatures above the phase transition of pure DMPC decreased, with the effect of these changes being greater after the addition of dStigS. These results are consistent with fluorescence polarization experiments and indicate that dStigS compounds are more effective than dStigC in increasing the conformation of the hydrocarbon chain in the liquid crystalline state of the DMPC membrane.

**Figure 9:**
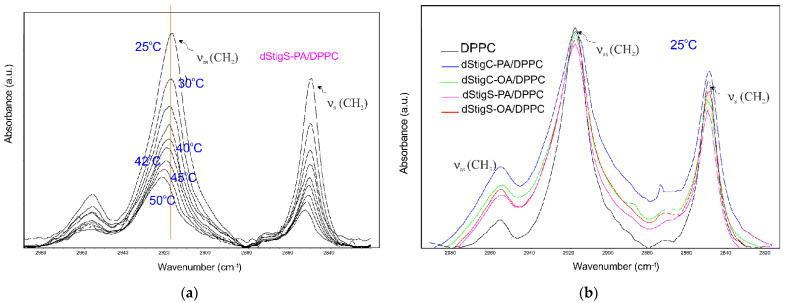
Infrared spectra of hydrated dStigS-PA/DPPC bilayer in the hydrocarbon chains region at different temperatures (**a**) and comparison of the spectra from the hydrophobic region of the bilayer of all tested compounds at the same temperature, in the gel phase (**b**); n_dStigMAs_/n_DPPC_ = 0.20.

Differences in the effect of dStigMAs on the vibrational frequencies of hydrocarbon chains of bilayers depending on the structure of the compound are clearly visible for the dStigMAs/DPPC system. Selected ATR-IR spectra of hydrated dStigS-PA/DPPC bilayer in the hydrocarbon chains region at different temperatures are shown in Figure 9a,b compares the spectra from the hydrophobic region of the bilayer of all tested compounds at the same temperature, in the gel phase.

**Figure 10:**
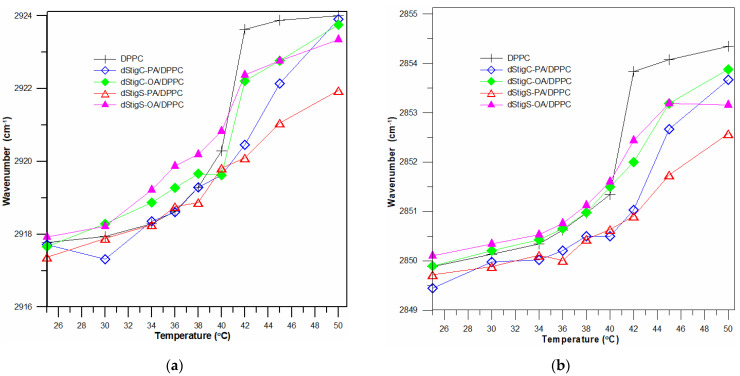
Dependence of vibration frequency on system temperature of the CH_2_ asymmetric (**a**) and symmetric (**b**) stretching mode in the presence and absence of dStigMAs for DPPC bilayer; n_dStigMAs_/n_DPPC_ = 0.20.

Whereas in Figure 10, the temperature-dependent changes in the vibration frequency of hydrocarbon chains for DPPC and for the dStigMAs/DPPC system are compared. Similar to DMPC membranes, we also observe the ordering effect that dStigMAs exert on the bilayer. The changes in frequency oscillations vary depending on the type of linker and the chain of modified glycerols. The compounds dStigC-PA and dStigS-PA cause a shift in the temperature of the main phase transition, changing, in particular, the dStigS-PA nature of the transition. The differences are more pronounced depending on the type of chain (Figure 10).

## 4. Discussion

The subject of our research presented in this manuscript are four acylglycerols containing two stigmasterol residues at *sn*-1 and *sn*-2 or *sn*-2 and *sn*-3 positions and the palmitic or oleic acid attached to *sn*-3 or *sn*-1 position (dStigMAs). The stigmasterol molecule has been attached using succinyl or carbonate linker. In earlier work, the physicochemical properties of dStigMAs were determined and their biological activity and stability were evaluated. Cytostatic activity was found to be dependent on the structure of the compound. In the present manuscript, we focus on biophysical studies aimed at verifying the effect of new distigmasterol-modified acylglycerols on the fluidity and phase transitions of lipid model membranes using calorimetry and spectroscopy methods. Measurements were carried out both in the gel phase and in the liquid crystalline phase of the lipid membrane. Physicochemical properties such as fluidity and the T_m_ are important parameters of the membranes affecting their stability and, consequently, their practical application as carriers of active substances.

Among sterols, cholesterol is the best known and the most commonly used to regulate membrane fluidity [24,25,28]. However, studies indicate that plant sterols also affect the structural properties and stability of the membranes [26,27,29] but their impact is different compared to cholesterol. Tamai at al. [26] found that stigmasterol has a stronger effect of disrupting chain packing in the gel state and a weaker effect of suppressing chain melting compared to cholesterol. Importantly, studies on the effects of stigmasterol as well as cholesterol on the physicochemical properties of membranes involve the free form of the molecule integrated into the lipid bilayer. In our study, we focused on molecules in which two stigmasterol residues are linked to acylglycerol by carbonate or succinyl linker. The purpose of our study was to compare the effects of these four compounds on the thermotropic parameters of lipid model membranes.

The fluorimetric method showed that dStigMAs change the fluidity and the T_m_ of both model membranes. A slight increase in membrane fluidity was observed in the gel phase, and a significant increase in membrane stiffness in the liquid crystal phase compared to the control was found. We also found a similar nature of changes for free stigmasterol (data shown in Figure S2). At the same time, the magnitude of these changes was smaller compared to dStigMAs at liquid phase, and greater at gel phase of lipid model membrane. Interestingly, for the membrane formed with DMPC in both the gel and liquid-crystalline phases, dStigMAs caused an increase in membrane stiffness. The value of anisotropy was higher compared to the control for all tested compounds. However, there were differences in the effect of dStigMAs on the fluidity of the bilayer depending on the type of chain and, to a lesser extent, on the linker, which could be presented in the following order: dStigS-PA > dStigS-OA and dStigC-PA > dStigC-OA and dStigS-PA > dStigC-PA. Moreover, in our earlier work, we showed that acylglycerols containing myristic acid residues and one stigmasterol molecule significantly increase the order in the hydrophilic region of the membrane; moreover, they cause a decrease in membrane fluidity and increase of T_m_ in the hydrophobic region [15]. Our results are in line with other authors who suggest that stigmasterol affects the thermotropic parameters of lipids, their ordering and the fluidity and hydration near the polar group of liposomes formed from DMPC [30]. These authors showed that the effect of stigmasterol on the physicochemical parameters of the membrane depends on the concentration of stigmasterol used. The effect was opposite for low concentrations compared to high concentrations of stigmasterol. Such an effect was not observed in our spectrofluorimetric studies. An increase in the concentration of dStigMAs in the bilayer had the effect of increasing changes in membrane fluidity. However, we found a very interesting effect using the calorimetric method.

Thermotropic parameters of model membranes formed from DPPC and DMPC with the tested compounds were analyzed using differential scanning calorimetry (DSC). All dStigMAs cause the elimination of the pretransition, reduce the cooperativity of the main phase transition and affect the T_m_. However, changes in the temperature of the main transition varied depending on the type and concentration of the compound. In general, dStigS-PA and dStigC-PA did not cause a decrease in T_m_, over the entire range of concentrations tested, while dStigS-OA at lower concentrations in the membrane affected a shift of T_m_ toward lower temperature values (about 0.5 °C). In contrast, an increase in the concentration of dStigS-OA caused the half-width of the peak to increase greatly and T_m_ to shift toward higher values. A slightly different effect on the main transition temperature depending on the type of membrane was observed only for dStigC-OA. For MLVs formed with DPPC, T_m_ under the influence of the compound practically does not change, while MLVs with DMPC decrease. A shift in the temperature of the main phase transition toward lower values is observed mainly for dStigS-OA and dStigC-OA, i.e., compounds containing an oleic acid, which is associated with a lower ordering in the gel phase of the hydrocarbon chains and a slight reduction in the temperature of the main phase transition. In addition, the asymmetric shape of the transition suggests the formation of domains containing an unbalanced amount of dStigMAs. A key role is played by stigmasterol, which acts as a separator by reducing interactions of acyl chains of phospholipids. Tamai et al. suggest that DPPC and stigmasterol are immiscible in the bilayer. Stigmasterol tends to form a complex consisting of one molecule and six DPPC molecules with which they explain the need for a higher concentration of stigmasterol compared to cholesterol to level the main phase transition. This is attributed to the presence of an additional side chain at the C24 position in the molecule. An increase in the concentration of compounds results in an increase in the content of stigmasterol molecules, the presence of which reduces van der Waals interactions between chains, decreasing the enthalpy of the transition. An increase in the concentration of compounds and thus stigmasterol causes the disappearance of the sharp phase transition. In the liquid crystalline state, stigmasterol causes a decrease in the average surface area per lipid near the membrane surface. To sum up, the most visible changes were observed for dStigS-PA and dStigS-OA, which at higher concentrations caused increase of the T_m_. Moreover, the enthalpy and half-width of the transition decrease significantly, which may indicate a change in lipid membrane fluidity. These results are in agreement with those of the fluorimetric method and are similar to the results presented in our earlier work [15].

The results obtained by the FTIR method also confirm that the presence of dStigMAs affects the fluidity of the lipid bilayer in the hydrophobic region and the T_m_ temperature of DMPC and of DPPC. The degree of these changes depends on the type of compound used. The presence of acylglycerols containing oleic acid (dStigC-OA) above the T_m_ temperature causes slight shifts toward higher values of wavenumbers, while for acylglycerol containing palmitic acid we observed slight shifts toward lower values (dStigC-PA) for DMPC membranes. However, for DPPC membranes, the effects are somewhat different. DStigC-OA and dStigS-OA increase the fluidity of the bilayer in the gel phase and increase the stiffness in the liquid crystalline phase, dStigS-PA and dStigC-PA decrease the fluidity of the hydrophobic region in the liquid crystalline phase, but do not increase the fluidity at temperatures below T_m_ of DPPC.

Rudzińska et al. [10] showed that the type of linker connecting stigmasterol to acylglycerol and the heating temperature affected the cytotoxicity and genotoxicity of the obtained dStigMAs. They found that acylglycerols possessing succinyl linker had no cytotoxic activity against normal human cells and had high thermooxidative stability. Our studies show that dStigMAs possessing succinyl linker, particularly dStigS-OA, resulted in increased gel-phase fluidity and rigidity of the DMPC and DPPC bilayers of the model membrane. It may be presumed that the increased cytotoxicity of dStigMAs with carbonate linker compared to dStigMAs with succinyl linker is due to the fact that dStigS have a bilayer-stabilizing effect. They prevent rapid chain melting, thus leveling the phase transition effect. Taking everything into account, we can conclude that the studied hybrids of acylglycerols with stigmasterol have a beneficial effect on improving the physicochemical parameters of model membranes, which has important implications for membrane stability. The results obtained are the basis for research on the practical use of these compounds. 

## 5. Conclusions

In this study, we compared the effects of four acylglycerols containing two stigmasterol residues and the palmitic or oleic acid on selected physicochemical properties of a model lipid membrane. Using DSC and IR and fluorescence spectroscopy methods, the effect of tested compounds on main phase transition of lipid bilayers and fluidity of DPPC and DMPC model membranes were determined. Measurements were made in the gel and in the liquid crystalline phase of the lipid membrane.

The use of complementary methods allowed us to conclude that all acylglycerols studied cause an increase in the rigidity of the membrane in the liquid crystalline phase. The intensity of these changes depends on the content of the studied compounds in the membrane, but above all, their structure is decisive. Acylglycerols with a succinyl linker affect the decrease in the cooperativity of the main phase transition, with an increase in the concentration of the compound to a greater extent compared to acylglycerols with a carbonate linker. In addition, the type of hydrocarbon chain matters, as shown by changes in anisotropy and IR studies.

Considering the beneficial effect of stigmasterol and the stabilization of model lipid membranes in the presence of the tested compounds, distigmasterol-modified acylglycerols, especially those possessing succinyl linker, are good candidates for functional foods. In particular, as demonstrated by the co-authors of this paper, acylglycerols with stigmasterol attached by succinyl linker are characterized by high thermooxidative stability and have no effect of cytotoxicity and genotoxicity on normal human cells.

The results provide a starting point for further research on the use of distigmasterol-modified acylglycerols in the design of dietary ingredients, such as liposomes carrying health-promoting phytosterols.

## Data Availability

The data presented in this study are available on request from the corresponding author.

## Supplementary Materials

The following supporting information can be downloaded at: https://www.mdpi.com/article/10.3390/membranes12111054/s1, Figure S1: Calorimetric curves of DMPC and dStigMAs /DMPC multilamellar liposomes (molar ratio of dStigMAs /DPPC = 0.2); Figure S2: Values of anisotropy of DPH probe fluorescence as a function of temperature for the control (DPPC) and a mixture of DPPC and stigmasterol (Stig) at molar ratios of Stig/DPPC: 0.20; 0.10; 0.05; 0.02 and 0.01.
