# Supplementary material for: Effect of Distigmasterol-Modified Acylglycerols on the Fluidity and Phase Transition of Lipid Model Membranes
Source: Membranes (Basel). 2022 Oct 27;12(11):1054. doi: 10.3390/membranes12111054 (PMC9698068; doi:10.3390/membranes12111054)
Supplement: Supplementary file 1 [file membranes-12-01054-s001.zip › membranes-1948101-supplementary.pdf]

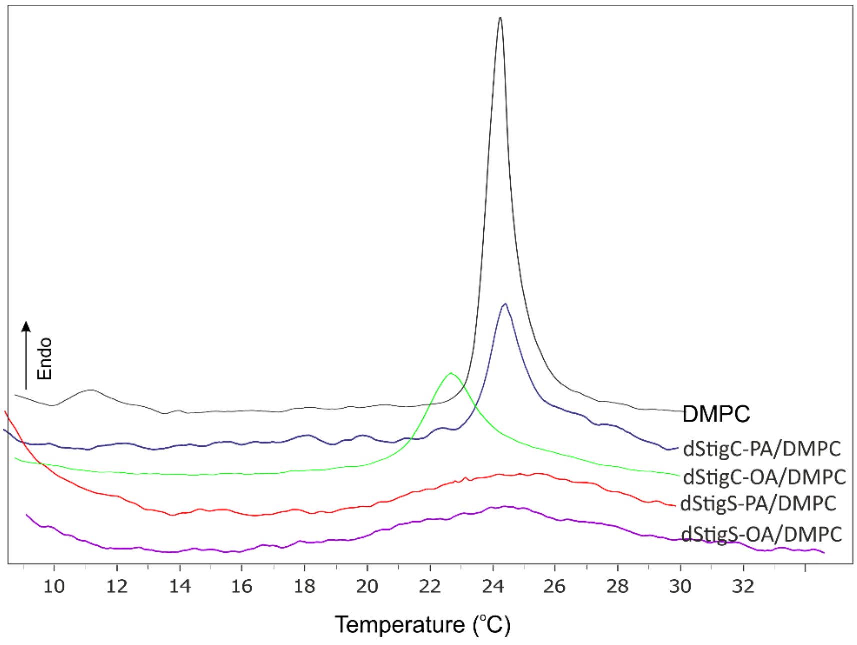

Figure S1. Calorimetric curves of DMPC and dStigMAs /DMPC multilamellar liposomes (molar ratio of dStigMAs /DPPC = 0.2)

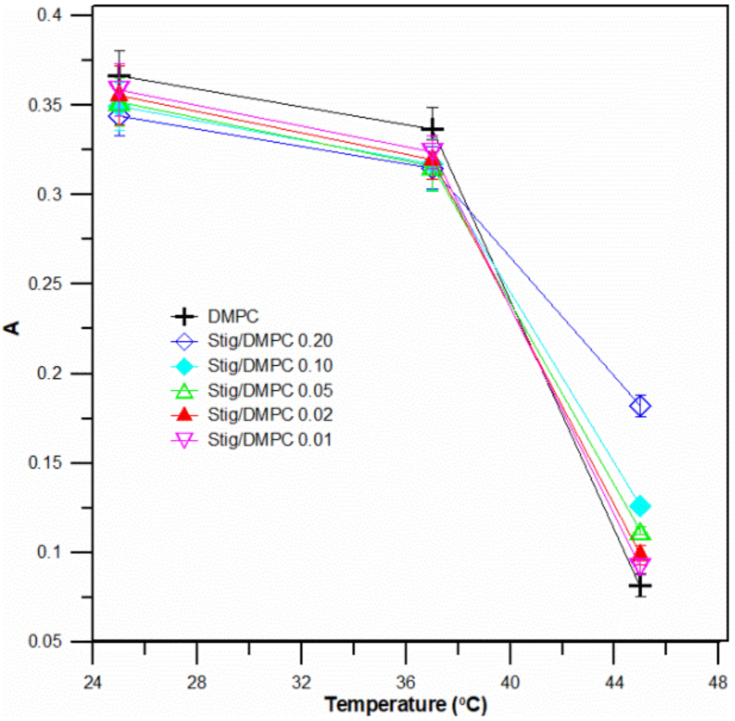

Figure S2. Values of anisotropy of DPH probe fluorescence as a function of temperature for the control (DPPC) and a mixture of DPPC and stigmasterol (Stig) at molar ratios of Stig/DPPC: 0.20; 0.10; 0.05; 0.02 and 0.01.
